# Genome-Wide Identification, Characterization and Expression Analysis of Soybean *CHYR* Gene Family

**DOI:** 10.3390/ijms222212192

**Published:** 2021-11-11

**Authors:** Bowei Jia, Yan Wang, Dajian Zhang, Wanhong Li, Hongli Cui, Jun Jin, Xiaoxi Cai, Yang Shen, Shengyang Wu, Yongxia Guo, Mingzhe Sun, Xiaoli Sun

**Affiliations:** 1 Crop Stress Molecular Biology Laboratory, College of Agriculture, Heilongjiang Bayi Agricultural University, Daqing 163319, China; jiabowei_paper@163.com (B.J.); 18645232886@163.com (Y.W.); wh10161122@163.com (W.L.); chl8023165@163.com (H.C.); jinjun19990421@163.com (J.J.); 18746616279@163.com (X.C.); 15603692106@163.com (Y.S.); m18846071994@163.com (S.W.); gyxia@163.com (Y.G.); 2 State Key Laboratory of Crop Biology, College of Agronomy, Shandong Agricultural University, Tai’an 271018, China; dajianzhang@sdau.edu.cn; 3 Key Laboratory of Germplasm Enhancement, Physiology and Ecology of Food Crops in Cold Region, Ministry of Education, Northeast Agricultural University, Harbin 150030, China

**Keywords:** CHYR, soybean, genome-wide identification, expression analysis, abiotic stress

## Abstract

The *CHYR* (CHY ZINC-FINGER AND RING FINGER PROTEIN) proteins have been functionally characterized in iron regulation and stress response in *Arabidopsis*, rice and *Populus*. However, their roles in soybean have not yet been systematically investigated. Here, in this study, 16 *GmCHYR* genes with conserved Zinc_ribbon, CHY zinc finger and Ring finger domains were obtained and divided into three groups. Moreover, additional 2–3 hemerythrin domains could be found in the N terminus of Group III. Phylogenetic and homology analysis of *CHYRs* in green plants indicated that three groups might originate from different ancestors. Expectedly, *GmCHYR* genes shared similar conserved domains/motifs distribution within the same group. Gene expression analysis uncovered their special expression patterns in different soybean tissues/organs and under various abiotic stresses. Group I and II members were mainly involved in salt and alkaline stresses. The expression of Group III members was induced/repressed by dehydration, salt and alkaline stresses, indicating their diverse roles in response to abiotic stress. In conclusion, our work will benefit for further revealing the biological roles of *GmCHYRs*.

## 1. Introduction

As one of the most widely grown crops in the world, soybean (*Glycine max*) provides an important source of plant-based protein and edible oil [1]. However, its yield and quality are enormously hindered by germplasm resources and diverse environmental factors, especially water deficiency, high salt, and alkaline [2]. Drought is one of the major natural disasters for world’s agricultural production. Globally, this extreme weather phenomenon has led to cereal loss of 1820 million Mg during the past four decades [3,4]. Soil saline–alkalization is another worldwide abiotic stress restraining land utilization, grain yield and local economic development. According to official statistics, more than 6% of the world’s soil resources are affected by saline and alkaline. Furthermore, continuous drought has a great influence on soil salinization and salt accumulation in root zone [5]. Utilization and management of the saline–alkaline soil is requisite to alleviate the ever-growing population’s demand for food. Consequently, it is meaningful to focus on uncovering the molecular mechanism of plant response to abiotic stress and cultivating crops with stress resistance.

The RING E3 (Really Interesting New Gene) proteins were found to play critical roles in abiotic stress response via protein ubiquitination degradation [6,7]. Previously, a C3H2C3 RING (Really Interesting New Gene) zinc finger domain containing protein from *Arabidopsis* was characterized and named as *MIEL1* (*MYB30-Interacting E3 Ligase1*) [8]. According to the conserved RING zinc finger domain, they were also called *CHYR* (*CHY ZINC-FINGER AND RING FINGER PROTEIN*) and *RZFP* (*RING ZINC-FINGER PROTEIN*) [9,10]. Protein sequence alignment has proved that *MIEL1*, *RZFP* and *CHYR* were in the same family, with conserved CHY zinc-finger, C3H2C3-type ring finger and rubredoxin-type fold domain [9]. In addition, when hemerythrin domains appeared in the N-terminus of CHY zinc finger domain, they were designated *BTS/BSTL* (*BRUTUS/BRUTUS-like*) in *Arabidopsis*, but *HRZ* (*Hemerythrin motif-containing RING-and Zinc-finger protein*) in rice [11,12,13,14]. Above all, we could uniformly define these proteins containing CHY zinc-finger, C3H2C3 -type ring finger and rubredoxin-type fold domain as the *CHYR* family.

Increasing evidence has shown the diverse roles of *CHYR* genes in plant growth, development and stress responses. MIEL1 was first found to control protein stability of MYB96 and MYB30 in balancing cuticular wax biosynthesis and defense [8,15,16]. *AtCHYR1* was reported to enhance ABA and drought responses by elevating ROS production and stomatal closure [9]. Homologous gene of *Populus euphratica* (*PeCHYR1*) showed similar phenotypes, enhancing drought tolerance, stomatal closure, and H_2_O_2_ production [17]. However, overexpression of *OsRZF34* (*AtCHYR1* homologous gene in rice) enhanced stomatal opening, leaf cooling and ABA insensitivity [10]. *CHYR* proteins with 2–3 additional hemerythrin domains (also known as *BTS/BTSL/HRZ*) were found to regulate iron response in *Arabidopsis* and rice [11,12,18].

Though several *CHYR* genes have been identified with diverse names, they have not yet been systematically analyzed at the gene family level. In particular, their roles in soybean development and stress response have not been uncovered. Here, in this study, 16 *CHYR* genes were identified through an extensive search of soybean genome (Wm82.a2.v1). Furthermore, their chromosome localization, phylogeny, conserved domains and expression patterns, especially in response to abiotic stress were comprehensively analyzed. These results will provide valuable clues for further functional studies on *GmCHYR* genes and their potential roles in abiotic stress.

## 2. Results

### 2.1. Identification and Phylogenetic Analysis of CHYR Genes from Soybean and Arabidopsis

To identify soybean *CHYR* genes, protein sequences of published *Arabidopsis* *CHYRs* [8,9,11,12,19] were used to construct a Hidden Markov Model (HMM) [20]. Whole soybean and *Arabidopsis* protein sequences were downloaded from Phytozome to carry out the local search. Finally, 16 soybean and 7 *Arabidopsis CHYR* genes were identified. The 23 proteins were proven to contain at least three conserved domains, including CHY zinc-finger (PF05495), C3H2C3-type ring finger (PF13639) and zinc ribbon domain (PF14599) according to Pfam and SMART analysis. For convenience’s sake, soybean *CHYR* genes were renamed *GmCHYR1* to *GmCHYR16* based on their order on the chromosomes, and genes from *Arabidopsis* were relabeled as *AtCHYR1* to *AtCHYR7*. Their involved information (including sequence length, hydropathicity, predicted protein location, classification, alternative name and functions) were listed in Table S1. As we could see from Table S1, amino acid numbers of *GmCHYRs* and *AtCHYRs* ranged from 234 to 1262. Their grand average of hydropathicity were all negative, indicating that *GmCHYRs* and *AtCHYRs* are hydrophilic proteins. Furthermore, these *CHYR* proteins were predicted to localize in the cytoplasm, or nucleus, or chloroplast. The cytoplasm and nucleus distribution of AtCHYR6/MIEL1 in *Arabidopsis* cells could support this result [8].

**Figure 1:**
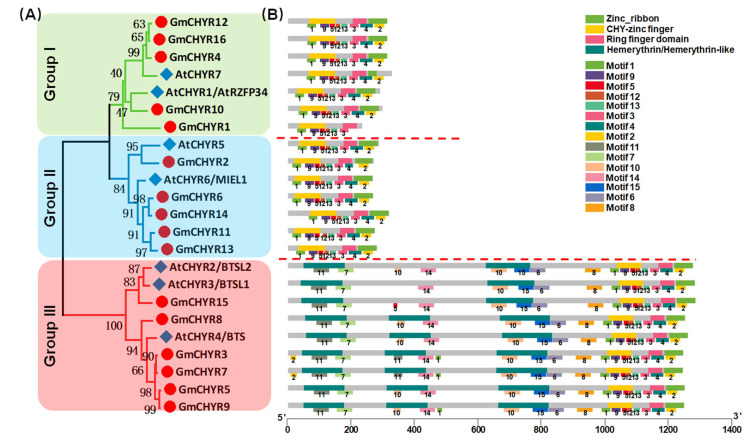
The phylogenetic tree and conserved domains and motifs analysis of *CHYR* genes in soybean and *Arabidopsis*. (**A**) Phylogenetic tree of soybean and *Arabidopsis* *CHYR* proteins, constructed by using MEGA 7.0 with the maximum-likelihood (ML) method under 1000 replications. (**B**) Conserved domains in GmCHYR proteins were identified by combining the SMART, PFAM, and NCBI CD database, represented by different colors. Green: Zinc_ribbon domain; Yellow: CHY-zinc finger domain; Pink: Ring finger domain; Dark green: Hemerythrin/Hemerythrin-like domain. The conserved motifs of GmCHYR proteins were analyzed by using the MEME tool. Schematic of the conserved domains and motifs were integrated by employing TBtools. The motif number was displayed below each motif.

To further investigate the phylogenetic relationship of GmCHYRs, their protein sequences were aligned with 7 AtCHYRs. All 23 *CHYR* proteins contained conserved CHY zinc-finger (PF05495), C3H2C3-type ring finger (PF13639) and zinc ribbon 6 domain (PF14599) (Figure S1). Then, a phylogenetic tree was generated basing on this multiple alignment by using MEGA 7.0 with the Maximum-Likelihood (ML) method with 1000 bootstrap replications. As shown in Figure 1A, soybean and *Arabidopsis* *CHYRs* could be classified into three groups according to their topological analysis and bootstrap values. In details, both Group I and Group II consisted of 5 *GmCHYRs* and 2 AtCHYRs. The rest, 6 *GmCHYRs* and 3 AtCHYRs, were allocated to Group III.

Furthermore, their conserved domains and motifs were analyzed. As expected, all 16 *GmCHYRs* and 7 *AtCHYRs* contained CHY zinc-finger, C3H2C3-type ring finger and zinc ribbon (Figure 1B). Besides, there were 2-3 hemerythrin domains in the N terminus of Group III members. Group III members were also called *BTS/BTSL* in *Arabidopsis*, and *HRZ* in rice [12,18]. This is consistent with former reported results that there were 2 *BTSL* (*AtCHYR2/3*) and 1 *BTS* (*AtCHYR4*) in *Arabidopsis* [12]. All of them have been reported to regulate iron homeostasis [11]. Meanwhile, we employed the MEME program to predict conserved motifs (Figure 1B). In accordance with conserved domains, *GmCHYRs* within each group displayed similar motif distribution. Among the detected 15 motifs, motif 1, 5, 9, 12 in the N terminus made up CHY-zinc finger. Motif 3 and 4 formed the Ring finger domain. Motif 2 served as Zinc_ribbon domain. Additionally, hemerythrin domain of Group III members constitutes of motif 7, 10, 11, 14, 15. Additionally, a conserved motif 6 and 8, which was closely to hemerythrin domain, could be found in Group III members. However, their function still needs further investigation.

### 2.2. Identification and Classification of CHYR Members in Green Plants

Above results showed that only Group III members contained 2–3 additional hemerythrin domains in the N terminus, which are of great importance in regulating iron homeostasis. We wondered whether Group III *CHYR* proteins gained these hemerythrin domains during evolution, or Group I and II lost these domains. Therefore, the local proteome sequences of 21 representative plant species, including Dicots, Monocots, Basal Angiosperms, Pteridophyta, Bryophyta, Chlorophyta and Gymnosperm were searched to identify potential *CHYR* genes by using the former *Arabidopsis* HMM. At last, a total of 107 nonredundant sequences were obtained from 21 detected plant species (Table 1 and Table S2). Pfam and SMART were further used to detect the three conserved domains for *CHYR* proteins, including CHY zinc-finger domain, C3H2C3-type ring finger domain and zinc ribbon domain.

**Figure 2:**
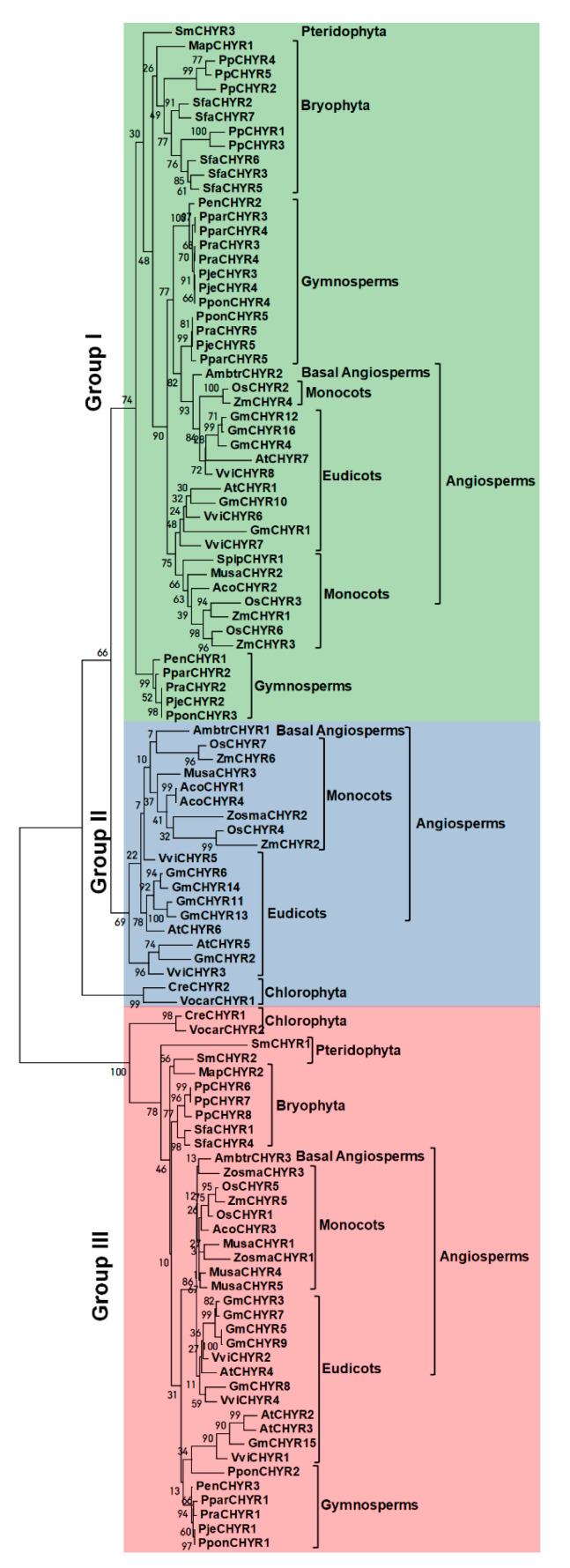
The Maximum-likelihood phylogenetic tree of *CHYR* genes in green plants. One hundred and seven *CHYR* protein sequences from 21 detected plant species were aligned with ClustalW and a phylogenetic tree was generated by using MEGA7 with the maximum-likelihood method under 1000 replications. The tree was divided into three groups with green shadow in Group I, blue shadow in Group II, and red shadow in Group III. Confidence values were listed on each node.

To explore their evolutionary relationship, 107 *CHYR* members were aligned using ML (Maximum-likelihood), NJ (Neighbor-joining), and ME (Minimum-evolution) methods to construct unrooted phylogenetic trees based on their protein sequences (Figure 2, Figure S2 and Figure S3). As the three phylogenetic trees depicted, three methods presented a similar topology. According to their evolutionary relationship, 107 *CHYR* members could be further divided into three groups (Group I, II, III) as well. Though Group I and Group II were clustered together, *CHYR* members from Bryophyta, Pteridophyta and Gymnosperms could be only found in Group I, implying the possibility of gene acquisition during evolution. From this result, we speculated that Group II might appear after Group I. Group III did coexist with the other two groups, but was far away from the others in topology, which indicated that they might come from different ancestors. Interestingly, there were only 4 *CHYR* members in Chlorophyta, two of them were from *Chlamydomonas reinhardtii*, the others were from *Volvox carteri*. While *CreCHYR2* and *VocarCHYR1* were clustered with Group I and Group II, *CreCHYR1* and *VocarCHYR2* were grouped together Group III, indicating the existence of *CHYR* members throughout green plants evolution. Previous study has reported the up regulation of *CreCHYR1* under iron deficiency [21], suggesting the conserved role of Group III members in iron regulating. The above findings implied the early emergence of *CHYR* members and their persistence in the evolution of green plants.

### 2.3. Homology Analysis of CHYR Genes from Soybean and Arabidopsis

**Figure 3:**
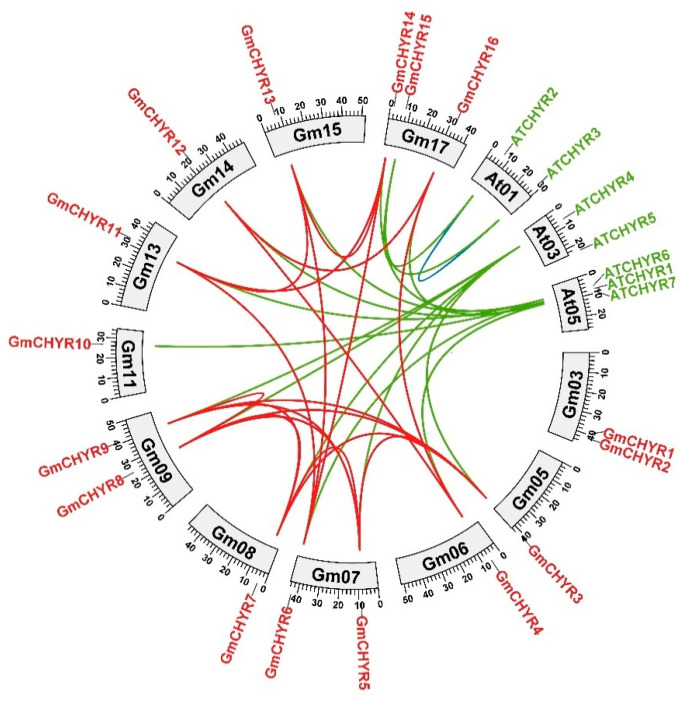
Chromosomal distribution and homology analysis of *CHYR* genes in the genomes of soybean and *Arabidopsis*. Paralogous and orthologous *CHYR* genes were mapped onto soybean and *Arabidopsis* chromosomes. Red lines connected soybean paralogous genes. Green lines indicated orthologous genes between *Arabidopsis* and soybean. Blue lines connected *Arabidopsis* paralogous genes.

According to their phylogenetic relationship, the number of *GmCHYRs* is more than twice that of *AtCHYRs*. Particularly, *GmCHYRs* appeared in pairs. The big genome size and whole genome duplication might be two critical reasons for gene expansion [22], such as gene duplication in soybean *LRR-RLK* genes [23]. The homologous relationship of *GmCHYRs* and *AtCHYRs* was further analyzed by comparing *G. max* and *A. thaliana* genomic sequence through OrthoVenn2 [24]. As depicted in Figure 3, 15 orthologous gene pairs were identified from *Arabidopsis* and soybean (green line in Figure 3). Nineteen paralogous gene pairs were characterized from soybean (red line in Figure 3), but only one paralogous gene pair exist in *Arabidopsis* (blue line in Figure 3), which might be derived from gene expansion during whole genome duplication occurred in soybean, or gene loss in *Arabidopsis* [25].

To trace their duplication time, *Ka* (non-synonymous rate), *Ks* (synonymous rate) and *Ka/Ks* ratios of 19 soybean paralogous genes were analyzed (Table S3). All *Ka/Ks* ratio of *GmCHYRs* were less than 1, varied from 0.12 to 0.4, indicating that they have undergone strong purify selection. Furthermore, their duplication time was calculated. The duplication time of Group I members varied from 9.5–43.6 Mya (million years ago) and Group II was around 11.5–46.4 Mya. This period is consistent with the latest twice whole genome duplication of soybean [25]. However, the duplication time of *GmCHYR3/GmCHYR8*, *GmCHYR5/GmCHYR8*, *GmCHYR7/GmCHYR8*, *GmCHYR8/GmCHYR9* pairs in Group III were greater than 155.6 Mya, which was just in line with the specific γ duplication of dicotyledon [25]. These results uncovered that *GmCHYR* expansion derived from whole genome duplication, resulting in conserved domains and motifs.

### 2.4. Expression Pattern of Soybean CHYR Genes in Different Tissues and Organs

**Figure 4:**
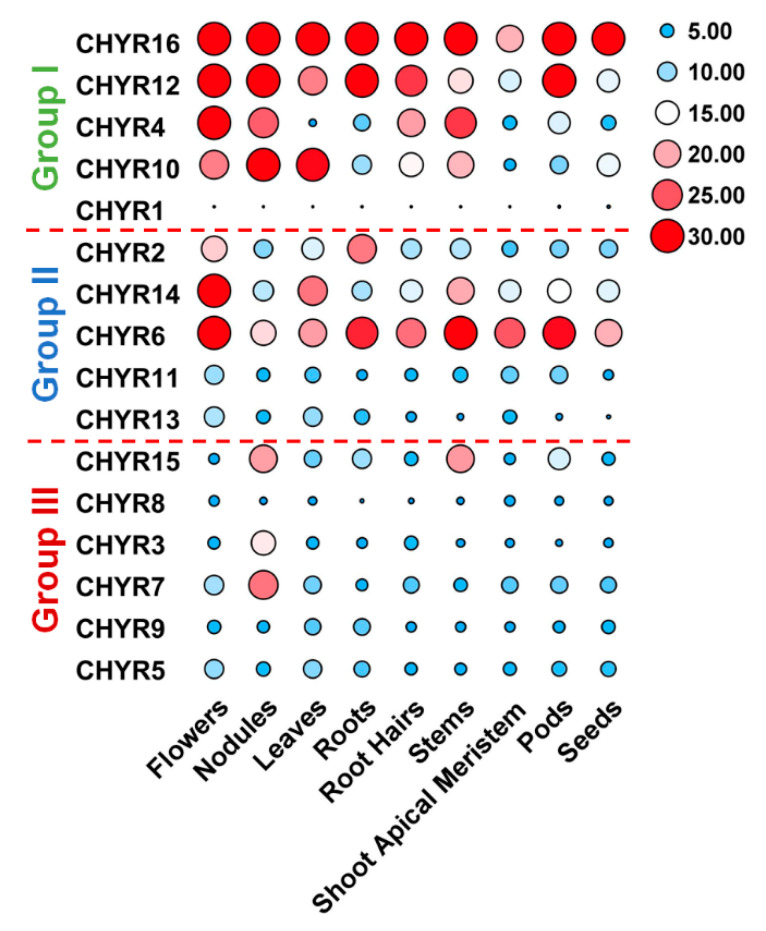
Tissue expression profiles of *GmCHYRs* in soybean. The transcriptional levels of *GmCHYR* genes in nine tissues/organs of soybean were analyzed based on published data collected in Phytozome. A heatmap were generated by TBtools. Five to thirty were artificially set with the color scale limits according to their expression values. The color scale shows increasing expression levels from blue to red.

To further look into *GmCHYRs* roles in soybean development, their expression profiles were analyzed based on published data of nine tissues/organs collected in Phytozome, including flowers, nodules, leaves, roots, root hairs, stems, shoot apical meristem, pods, and seeds [26]. As Figure 4 depicted, except that *GmCHYR1* showed almost no expression, the rest 15 *GmCHYRs* displayed specific expression across nine detected tissues/organs. Compared with Group III, Group I and II members were more likely to be expressed in all detected tissues/organs and had much higher expression values. This suggested their potential roles in soybean growth and development. Group II genes showed relative higher expression in the flowers, suggestive of their roles in reproduction. In particular, paralogous gene *GmCHYR6* and *GmCHYR14* were all highly expressed in nine detected tissues/organs. However, Group III members preferred to be expressed in nodules, indicating their roles in nitrogen fixation. In general, paralogous gene *GmCHYR4/12/16*, *GmCHYR6/11/13/14* and *GmCHYR3/7* shared similar expression patterns. *GmCHYR5/8/9* were also paralogs of *GmCHYR3/7*, but they displayed opposite expression from *GmCHYR3/7*. This might result from some special regulatory elements, or modification in their promoters, or just functional segregation during evolution.

### 2.5. Transcription Patterns of GmCHYRs in Response to Dehydration, Saline, Alkaline Stresses

**Figure 5:**
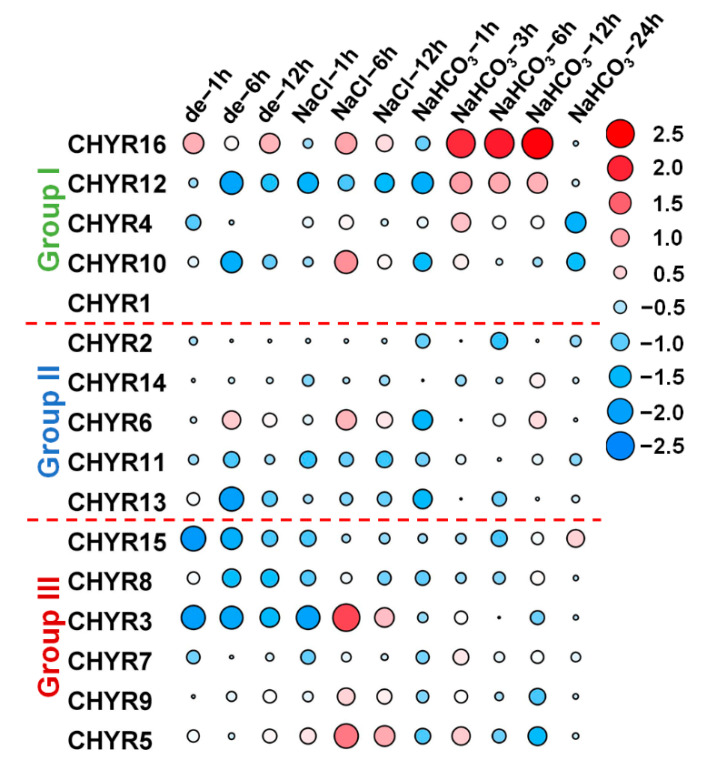
Expression profiles of *GmCHYRs* under dehydration, salt, and alkaline stress. The transcriptional levels of *GmCHYR* genes in response to dehydration (abbreviated as de), salt (100 mM NaCl) and alkaline (50 mM NaHCO_3_) stresses were investigated based on the published transcriptome data. The expression of *GmCHYR* were normalized by TBtools. According to the normalized value, −2.5 to 2.5 was artificially set with the color scale limits. The color scale shows increasing expression levels from blue to red. The differentially expressed genes (DEGs) were highlighted by red (up-regulation) and blue (down-regulation).

To uncover the roles of *GmCHYRs* in response to abiotic stress, their transcriptome data under different stress treatments were analyzed by using published database (including drought, salt (GSE57252) [27] and alkaline [28]). In accordance with tissue expression data, *GmCHYR1* showed little expression in root and its expression values were zero under abiotic stresses (Figure 5). As we could see from Figure 5, *GmCHYRs* in roots displayed various response strategies to diverse abiotic stresses. Group I members were involved in all detected stresses, and they were dramatically increased under alkaline stress. Among Group I, *GmCHYR16* was significantly up regulated by three stresses. However, its paralogous gene, *GmCHYR12* were only induced by alkaline stress. Another paralogous gene *GmCHYR4* exhibited similar expression patterns with *GmCHYR10*, which were repressed by dehydration, but induced by saline and alkaline stresses. Group II members exhibited similar expression profiles under abiotic stresses. Besides *GmCHYR6* showed increased expression under three detected stresses, most of Group II were increased under alkaline stress, but decreased under dehydration and saline stresses. Compared with Group I and II, genes from Group III were dramatically up regulated by salt stresses. Paralogous gene *GmCHYR3/7/5/9* exhibited similar expression profiles. However, the expression of *GmCHYR8* and *GmCHYR15* were repressed by three stresses. Above all, Group I members might play vital roles in dehydration, salt and alkaline stresses. Group II and III members were participated in salt and alkaline response.

### 2.6. qRT-PCR Verification of GmCHYRs under Dehydration, Saline and Alkaline Stresses

**Figure 6:**
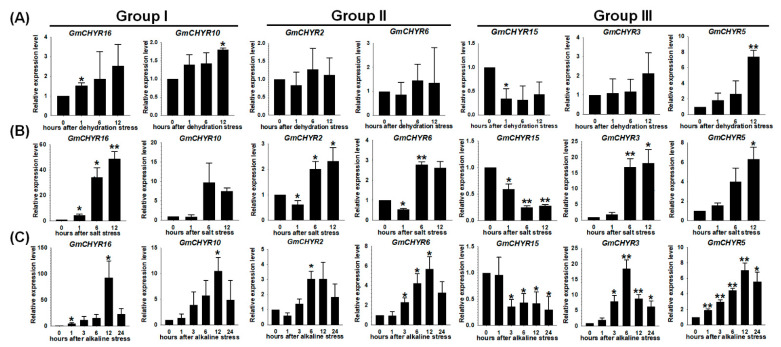
Quantitative real time-PCR expression analysis of 7 selected *GmCHYRs* under dehydration (**A**), salt (**B**), and alkaline (**C**) stresses. The soybean ubiquitin 3 gene was used as reference control. Their transcript levels were normalized to 1 at 0 h. Other points were presented as relative fold changes compared with 0 h. Error bars represented the standard deviation. Asterisk indicated significant differences among mean values compared with 0 h (Student’s *t*-test, * *p* < 0.05, ** *p* < 0.01). The results were based on three replicates in three independent experiments.

To confirm that they were indeed involved in the three stresses, the expression of 7 genes from three groups under dehydration, salt and alkaline stresses were validated by qRT-PCR, including *GmCHYR16/10* (Group I), *GmCHYR2/6* (Group II), and *GmCHYR3/5/15* (Group III) (Figure 6). These 7 genes were chosen according to their homology relationship and specific stress expression patterns. Their expression trends were basically consistent with transcriptome results. *GmCHYR16* (Group I) was dramatically induced more than 93-fold under alkaline stress and 49-fold under saline stress, respectively. Under dehydration stress, the expression fold change of *GmCHYR16* was only 2.5. This suggested the vital roles of *GmCHYR16* in salt and alkaline stress response. *GmCHYR10* (Group I) displayed similar expression patterns with *GmCHYR16*. In accord with transcriptome results, the expression level of *GmCHYR10* was lower than that of *GmCHYR16*. Moreover, *GmCHYR2/6* (Group II) were induced by salt and alkaline stresses, but did not respond to dehydration. *GmCHYR3/5* (Group III) were dramatically induced by three stresses, while *GmCHYR15* (Group III) depicted down-regulated trends under three stresses, suggesting their functional differentiation during evolution.

## 3. Discussion

For a long time, soybean is one of the staple crops and the most important legume in the world, which provides a major source of vegetable protein and edible oil [29]. Nowadays, with the continuous improvement of living level, the aging, the change of population structure and the fast development of stock farming, the consumption of protein and oil products has increased day by day, driving the consumption of soybean. However, such as other crops, soybean yield is restricted by natural environmental conditions. Previous studies have shown that adverse stresses had negative effects on plant growth and development, including abnormal metabolism, protein misfolding and so on [30]. Molecular design breeding is one of the effective methods to improve crops stress resistance. The key is to mine stress-resistance genes.

Previously, *CHYR* genes with three conserved domains (CHY-zinc finger, Ring finger domain and Zinc_ribbon domain) have been reported in *Arabidopsis* [9,11], rice [10] and *Populus* [17] in response to adverse stresses. However, little was known about them in soybean. In this study, a total of 16 *CHYR* genes were identified through searching against the released genome database of *G. max* [26] by using *AtCHYRs* protein sequences as queries [9,11]. According to their phylogenetic analysis, *CHYRs* could be classified into three groups (Figure 1A, Figure 2, Figure S2 and Figure S3). This result was further supported by conserved domain and motif distribution. An exploration of conserved domain and motif distribution confirmed that all GmCHYR proteins contained conserved CHY-zinc finger, Ring finger domain and Zinc_ribbon domain (Figure 1 and Figure S1). In addition, there were 2–3 hemerythrin domains in the N-terminus of Group III members (Figure 1 and Figure S1). The similar results had been previously reported in *Arabidopsis* [9,11], rice [10] and *Populus* [17]. Meanwhile, conserved motifs 6 and 8 were found in Group III members, between the CHY-zinc finger domain and hemerythrin domain (Figure 1), while its function was still unknown. The above results suggested that genes shared similar conserved domain and motif distribution within the same group.

As known to all, the conserved zinc finger domains consisted of several conserved cysteine and histidine residues (Cys - X2 - Cys - X (9–39) - Cys - X (1–3) - His - X (2–3) - Cys/His - X2 - Cys - X (4–48) - Cys - X2 - Cys) to bind zinc ions (Figure S1), or interact with partner proteins, or catalyze E3 ubiquitin ligase activity. For example, the RING domain of *AtCHYR1* (Group I) was essential for ubiquitin E3 ligase activity [9]. The CHY-zinc finger domain of *AtCHYR6/MIEL1* (Group II) was responsible for MYB96 interacting and degrading [16]. At the same time, Group III members containing hemerythrin domains could interact with transcription factor PYEL proteins and exert E3 ligase activity via the C-terminal RING domain [31]. The hemerythrin domain in plants was reported to play essential roles in iron binding and protein stability [13]. The removal of hemerythrin domain could make Group III *CHYR* (also termed as BTS) stable in the existence of iron and also complement hemerythrin-containing *CHYR* loss, while deletion of the RING domain could not [13,31]. *AtCHYR2/3/4* (Group III) and *OsCHYR1/5* (Group III) were reported to act as negative regulators in iron deficiency response [11,12,32]. However, E3 ubiquitin ligase activity and iron regulating function of *GmCHYRs* still need further experiments to validate.

It was worth noting what drove the difference between Group I/II and Group III in gene structure and physiological roles. Further phylogenetic analysis of *CHYRs* in green plants confirmed that *CHYR* genes appeared during green plant evolution (Figure 2). This was consistent with reported result that Group III members exist among photosynthetic organisms as well [13]. However, the divergence of Group I and Group II members might occur after angiosperm differentiation (Figure 2, Figure S2 and Figure S3). The number of *CHYR* family in *G. max* is more than two folds that of *Arabidopsis* (7 *CHYR* genes) and rice (7 *CHYR* genes), but is relative stable in detected species within Eudicots, Monocots, Gymnosperm, and Chlorophyta (Table 1, Table S1 and Table S2). A similar phenomenon has been found in other ubiquitin ligase families. For example, *U-box* [33], *HETC* (*homologous to the E6AP carboxyl terminus*) [34] family in soybean are more than twice that of *Arabidopsis* and rice. Whole genome duplication (WGD) is a key reason for gene expansion, gene loss and new functionalization. As previously reported, *G. max* has gone through three whole genome duplications (117, 59, and 13 Mya) [25], which might lead to the emergence of 19 paralogous gene pairs (Figure 3). A further *Ka/Ks* calculation also confirmed that purification selection was the main driving force in the evolution of *GmCHYRs* (Table S3).

In terms of GmCHYRs’ biological function, the expression of Group I and II members were much higher than that of Group III in the nine detected tissues/organs. Group I and II members were expressed in all tissues/organs, while Group III members preferred to be expressed in nodules (Figure 4). According to this result, we inferred that *GmCHYRs* might be involved in nitrogen-fixing genes via ubiquitination. Tissue-specific and stress expression pattern analysis of *GmCHYRs* were helpful to uncover their potential roles in physiology and development. Previously, studies about *CHYRs* were mainly focused on iron regulation. For example, *AtCHYR4* (*BTS*) and *AtCHYR2/3* (*BTSL1/2*) from Group III were induced by iron deficiency [12,19]. The expression of rice Group III *CHYR1/5* (also known as *HRZ1/2*) were up regulated by iron insufficiency [14]. Even Group III member of *C. reinhardtii*, *CreCHYR1* was up regulated by iron deficiency as well [21]. These results suggested the conserved roles of Group III members in iron regulating.

Our lab has long been committed to the study of crop abiotic stress response. The expression of *GmCHYRs* under salt, alkaline and drought stresses was further examined. According to the published transcriptome data, only 15 *GmCHYRs* (except for *GmCHYR1*) were detected and they were all up regulated/down regulated by dehydration, salt and alkaline stresses, suggesting their potential role in stress response (Figure 5). Due to gene homology, the expression of seven genes (*GmCHYR10/16* (Group I), *GmCHYR2/14* (Group II), and *GmCHYR3/5/15* (Group III)) from three groups were further confirmed by using qRT-PCR (Figure 6). We could confirm that the expression of *GmCHYR15* was repressed by dehydration, salt and alkaline stresses, while the expression of *GmCHYR3/5* was induced by these stresses by integrating transcriptome data and qRT-PCR. Though *GmCHYR3/5* and *GmCHYR15* belong to Group III, their opposite expression patterns might derive from functional differentiation during evolution or cis-regulatory elements in promoter regions. *GmCHYR10/16* (Group I) and *GmCHYR2/14* (Group II) were all significantly upregulated by salt and alkaline stresses, up to 93-fold, but only 2.5-fold under dehydration stress, indicating their special roles in salt and alkaline response. In particular, *GmCHYR16* might be a key regulator in salt and alkaline stress response.

Above all, our analysis of 16 *GmCHYRs* showed that they share highly conserved domains and residues, indicating their potential conserved structure and biochemical function. Most of *GmCHYRs* were found to respond to various stresses, especially Group I and II members might play positive regulators in abiotic stress response. However, their internal regulatory mechanisms are still misty. Whether *GmCHYRs* function as an E3 ubiquitin ligase also requires further experimental verification.

## 4. Materials and Methods

### 4.1. Identification of CHYR Genes from Green Plants

A Hidden Markov Model (HMM) basing on published *CHYR* protein sequences from *Arabidopsis* were constructed to search against 21 green plants genomes [8,9,11,12,18,19,20]. These genomes were collected from NCBI (https://www.ncbi.nlm.nih.gov/genome/, last accessed on 6 June 2020), Phytozome(https://phytozome.jgi.doe.gov/pz/portal.html, last accessed on 6 June 2020) [26] and Congenie (https://congenie.org/, last accessed on 10 June 2020) [35]. CHY zinc-finger domain (PF05495), C3H2C3-type ring finger domain (PF13639) and zinc ribbon domain (PF14599) of putative *CHYR* genes were then verified by using CD search (https://www.ncbi.nlm.nih.gov/Structure/cdd/wrpsb.cgi, last accessed on 10 June 2020), Pfam (http://pfam.xfam.org/, last accessed on 10 June 2020) [36] and SMART (http://smart.embl-heidelberg.de/, last accessed on 10 June 2020) [37] with default parameters.

### 4.2. Phylogenetic Relationship, Sequence Alignments and Protein Localization Analysis

The phylogenetic relationship of *CHYR* genes were analyzed by using MEGA 7 through Neighbor Joining (NJ), Maximum Likelihood (ML) and/or Minimum evolution (ME) methods with 1000 bootstrap. Multiple sequence alignments were carried out by using Clustal Omega (https://www.ebi.ac.uk/Tools/msa/clustalo/, last accessed on 1 July 2020) [38] under default parameters. Protein locations were predicted by integrating SoftBerry (http://linux1.softberry.com/all.htm, last accessed on 10 June 2020), PSORT (https://www.genscript.com/psort.html, last accessed 10 June 2020), and CELLO (http://cello.life.nctu.edu.tw/, last accessed on 10 June 2020).

### 4.3. Chromosomal Distribution, Homology and Motif Analysis

The position information of *CHYR* genes on chromosome were picked up from soybean and *Arabidopsis* annotation [39] by using TBtools [40]. Their homology was then analyzed via OrthoVenn2 (https://orthovenn2.bioinfotoolkits.net/home, last accessed on 7 June 2021) [24]. MEME (http://meme.nbcr.net/meme/cgi-bin/meme.cgi, last accessed on 28 June 2021) was further carried out to investigate their motif [41]. Finally, TBtools was applied to make these information visualization [40]. The *Ka* (non-synonymous rate), *Ks* (synonymous rate), and *Ka/Ks* ratios were calculated by using TBtools according to their coding sequence. The duplication time was calculated according to published method by using the following formula: Time = *Ks*/(2 × substitution rate) and the substitution rates of soybean and *Arabidopsis* are 6.1 × 10^−9^, and 1.5 × 10^−8^ site per year, respectively [42,43].

### 4.4. Expression Analysis of GmCHYR during Soybean Development and Response to Abiotic Stresses

The transcription data of *GmCHYR* from nine tissues/organs and under abiotic stresses (GSE57252 for drought and salt stress) [27,28] in soybean were collected from Phytozome, the 1KP Project (http://www.onekp.com/, last accessed on 6 July 2020) and NCBI GEO DataSets (https://www.ncbi.nlm.nih.gov/gds/, last accessed on 6 July 2020). Then, the correlation heatmap was analyzed by using TBtools [40].

### 4.5. Quantitative Real-Time PCR Analyses

Consistent with the transcription data, *G. max* seeds were cultured in distilled water for 1 day. Then, the swelled seeds were removed to a new Petri dish and covered with wet gauze. Once the roots grew to 1 cm, they were transferred into 1/4 Hoagland’s solution with 60% relative humidity, 24 °C, and 16 h light/8 h dark. When soybean reached the v1 stage (first trifoliolate stage), seedlings were transferred into 100 mM NaCl solution (salt stress) and 50 mM NaHCO_3_ (alkaline stress). For dehydration stress, soybean seedlings were removed from cultural solution and dried in air. Roots were harvested after 0 h, 1 h, 3 h, 6 h, 12 h, and 24 h. Three individual plants per point were used for each stress level.

Total RNA was extracted by using the TRIZOL reagent (Invitrogen, Waltham, MA, USA). Then, the cDNA was synthesized through the HiScript III RT SuperMix for qPCR (Vazyme, Nanjing, China). TransStart^®^ Top Green qPCR SuperMix (TransGen Biotech, Beijing, China) was used to perform quantitative real-time PCR (qRT-PCR). The relative expression levels were calculated according to the formula 2^−∆∆Ct^ [44]. The expression levels were normalized to 1 at 0 h. Then, the relative fold changes of other points were calculated compared with 0 h. Gene specific primers of *GmCHYRs* and the internal reference gene (*soybean ubiquitin 3*, *Glyma.20g141600*) [45] were listed in Table S4.

All of the above numerical data were subjected to statistical analyses using EXCEL 2010 and Prism 9 statistical software by Student’s *t*-test.

## Figures and Tables

**Table 1:** Overview of genes encoding *CHYR* proteins in plants.

Major Lineage	Species	Group I	Group II	Group III
Dicots	*Vitis vinifera*	3	2	3
	*Arabidopsis thaliana*	2	2	3
	*Glycine max*	5	5	6
Monocots	*Zea mays*	3	2	1
	*Oryza sativa*	3	2	2
	*Ananas comosus*	1	2	1
	*Musa acuminata*	1	1	3
	*Spirodela polyrhiza*	1	0	0
	*Zostera marina*	0	1	2
Basal angiosperms	*Amborella trichopoda*	1	1	1
Gymnosperm	*Pinus parviflora*	4	0	1
	*Pinus radiata*	4	0	1
	*Pinus jeffreyi*	4	0	1
	*Pinus ponderosa*	4	0	1
	*Picea engelmanii*	3	0	0
Pteridophyta	*Selaginella moellendorffii*	1	0	2
Bryophyta	*Marchantia polymorpha*	1	0	1
	*Physcomitrella patens*	5	0	3
	*Sphagnum fallax*	5	0	2
Chlorophyta	*Chlamydomonas reinhardtii*	0	1	1
	*Volvox carteri*	0	1	1

## Data Availability

All data are contained within the article or Supplementary Materials.

## Supplementary Materials

The following are available online at https://www.mdpi.com/article/10.3390/ijms222212192/s1.
