# Supplementary material for: Genome-Wide Identification, Characterization and Expression Analysis of Soybean CHYR Gene Family
Source: Int J Mol Sci. 2021 Nov 11;22(22):12192. doi: 10.3390/ijms222212192 (PMC8625759; doi:10.3390/ijms222212192)
Supplement: Supplementary file 1 [file ijms-22-12192-s001.zip › Supplementary Figure S1.pdf]

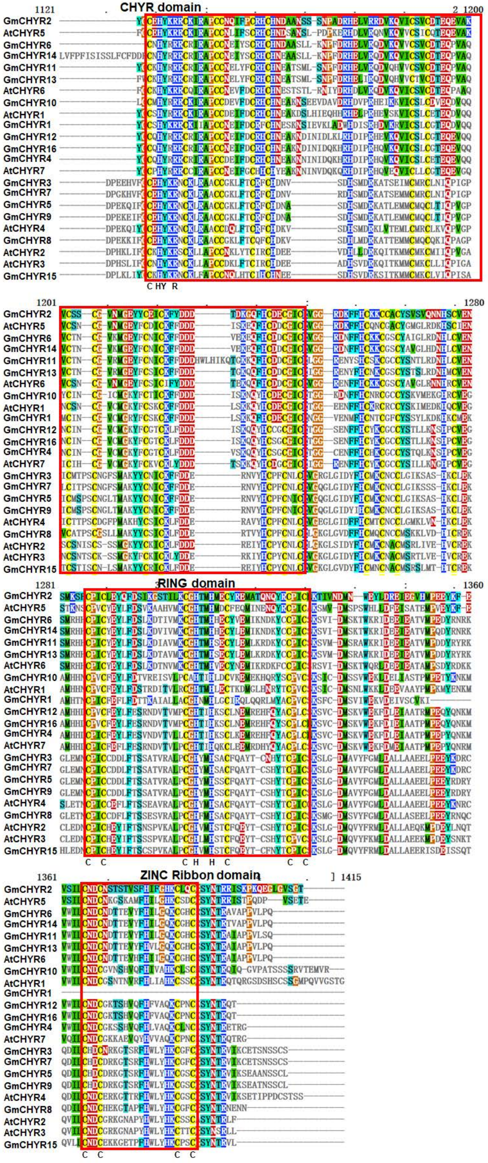
CHYR domain
1121
1200
GmCHYR2
AtCHYR5
GmCHYR6
GmCHYR14
GmCHYR11
GmCHYR13
AtCHYR6
GmCHYR10
AtCHYR1
GmCHYR1
GmCHYR12
GmCHYR16
GmCHYR4
AtCHYR7
GmCHYR3
GmCHYR7
GmCHYR5
GmCHYR9
AtCHYR4
GmCHYR8
AtCHYR2
AtCHYR3
GmCHYR15
C HY R
1201
1280
RING domain
1281
1360
C C C H H C C C
ZINC Ribbon domain
1361
1415
C C C C
