# Supplementary material for: Genome-Wide Identification, Characterization and Expression Analysis of Soybean CHYR Gene Family
Source: Int J Mol Sci. 2021 Nov 11;22(22):12192. doi: 10.3390/ijms222212192 (PMC8625759; doi:10.3390/ijms222212192)
Supplement: Supplementary file 1 [file ijms-22-12192-s001.zip › Supplementary Table S1 Features of CHYR genes identified in soybean and Arabidopsis.pdf]

Supplementary Table S1 Features of *CHYR* genes identified in soybean and *Arabidopsis*

No.	Genes name	Gene ID	Sequence length				GRAVY	Predicted location	Subfamily	Alternative names	Function	Named by
			DNA (bp)	mRNA (bp)	cDNA (bp)	protein (aa)						
1	*GmCHYR1*	*Glyma.03G185700*	2126	792	705	234	-0.276	Cytoplasm and Nucleus	Group I	None	None	This work
2	*GmCHYR4*	*Glyma.06G074300*	5818	1559	930	309	-0.562	Cytoplasm and Nucleus	Group I	None	None	This work
3	*GmCHYR10*	*Glyma.11G192900*	3766	1530	897	298	-0.300	Cytoplasm and Nucleus	Group I	None	None	This work
4	*GmCHYR12*	*Glyma.14G130700*	3793	1456	927	308	-0.540	Cytoplasm and Nucleus	Group I	None	None	This work
5	*GmCHYR16*	*Glyma.17G202700*	6292	3863	927	308	-0.606	Cytoplasm and Nucleus	Group I	None	None	This work
6	*GmCHYR2*	*Glyma.03G209900*	3587	1189	816	271	-0.565	Cytoplasm and Nucleus	Group II	None	None	This work
7	*GmCHYR6*	*Glyma.07G250900*	3495	1482	804	267	-0.479	Cytoplasm	Group II	None	None	This work
8	*GmCHYR11*	*Glyma.13G215600*	4089	1366	825	274	-0.479	Cytoplasm and Nucleus	Group II	None	None	This work
9	*GmCHYR13*	*Glyma.15G097300*	3854	1269	840	279	-0.396	Chloroplast	Group II	None	None	This work
10	*GmCHYR14*	*Glyma.17G023400*	3554	1698	960	319	-0.333	Cytoplasm and Chloroplast	Group II	None	None	This work
11	*GmCHYR3*	*Glyma.05G237500*	10976	4351	3711	1236	-0.332	Cytoplasm and Chloroplast	Group III	None	None	This work
12	*GmCHYR5*	*Glyma.07G093700*	10317	4466	3729	1242	-0.341	Cytoplasm and Chloroplast	Group III	None	None	This work
13	*GmCHYR7*	*Glyma.08G044700*	12059	4223	3705	1234	-0.342	Nucleus and Chloroplast	Group III	None	None	This work
14	*GmCHYR8*	*Glyma.09G115100*	11840	4156	3708	1235	-0.272	Cytoplasm and Nucleus	Group III	None	None	This work
15	*GmCHYR9*	*Glyma.09G182600*	11787	4516	3717	1238	-0.343	Nucleus and Chloroplast	Group III	None	None	This work
16	*GmCHYR15*	*Glyma.17G096900*	10682	4132	3789	1262	-0.345	Nucleus and Chloroplast	Group III	None	None	This work
17	*AtCHYR1*	*AT5G22920*	2275	1262	876	291	-0.648	Cytoplasm and Nucleus	Group I	None	None	This work
18	*AtCHYR7*	*AT5G25560*	3839	1455	987	328	-0.468	Cytoplasm and Nucleus	Group I	None	None	This work
19	*AtCHYR5*	*AT3G62970*	2291	1092	864	287	-0.451	Cytoplasm and Nucleus	Group II	None	None	This work
20	*AtCHYR6*	*AT5G18650*	2791	1480	804	267	-0.552	Cytoplasm and Nucleus	Group II	MIEL1 (MYB30-Interacting E3 Ligase1)	Regulators in controlling protein stability of MYB96 and MYB30 in cuticular wax biosyn thesis and defense	[8, 15, 16]
21	*AtCHYR2*	*AT1G18910*	5587	4064	3765	1254	-0.270	Cytoplasm and Nucleus	Group III	BTSL2	Redundantly as negative regulators of the Fe deficiency response	[11-13, 19]
22	*AtCHYR3*	*AT1G74770*	5264	3904	3780	1259	-0.334	Cytoplasm and Nucleus	Group III	BTSL1	Redundantly as negative regulators of the Fe deficiency	[11-13, 19]

											response	
23	*AtCHYR4*	*AT3G18290*	6516	4414	3765	1254	-0.350	Cytoplasm and Nucleus	Group III	BTS, EMB2454, zinc fnger protein-related	Redundantly as negative regulators of the Fe deficiency response	[11-13, 19]
