# Supplementary material for: Genome-Wide Identification, Characterization and Expression Analysis of Soybean CHYR Gene Family
Source: Int J Mol Sci. 2021 Nov 11;22(22):12192. doi: 10.3390/ijms222212192 (PMC8625759; doi:10.3390/ijms222212192)
Supplement: Supplementary file 1 [file ijms-22-12192-s001.zip › Supplementary Table S2 The basic information of CHYR genes from 20 species.pdf]

Supplementary Table S2 The basic information of *CHYR* genes from 20 species

<table>
<tr><th>Major lineage</th><th>Spieces</th><th>Abbr1</th><th>Abbr2</th><th>Gene Name</th><th>Gene ID</th><th>Group</th><th>Alternative name</th></tr>
<tr><td>Dicots</td><td>Vitis vinifera</td><td>Vitvi</td><td>V. vinifera</td><td>VviCHYR1</td><td>GSVIVG01008081001</td><td>III</td><td></td></tr>
<tr><td></td><td></td><td></td><td></td><td>VviCHYR2</td><td>GSVIVG01008317001</td><td>III</td><td></td></tr>
<tr><td></td><td></td><td></td><td></td><td>VviCHYR3</td><td>GSVIVG01010990001</td><td>II</td><td></td></tr>
<tr><td></td><td></td><td></td><td></td><td>VviCHYR4</td><td>GSVIVG01011864001</td><td>III</td><td></td></tr>
<tr><td></td><td></td><td></td><td></td><td>VviCHYR5</td><td>GSVIVG01017793001</td><td>II</td><td></td></tr>
<tr><td></td><td></td><td></td><td></td><td>VviCHYR6</td><td>GSVIVG01031467001</td><td>I</td><td></td></tr>
<tr><td></td><td></td><td></td><td></td><td>VviCHYR7</td><td>GSVIVG01033674001</td><td>I</td><td></td></tr>
<tr><td></td><td></td><td></td><td></td><td>VviCHYR8</td><td>GSVIVG01035743001</td><td>I</td><td></td></tr>
<tr><td></td><td>Arabidopsis thaliana</td><td>Ath</td><td>A. thaliana</td><td>AtCHYR1</td><td>AT5G22920</td><td>I</td><td>AtRZFP34[9]</td></tr>
<tr><td></td><td></td><td></td><td></td><td>AtCHYR2</td><td>AT1G18910</td><td>III</td><td>BTSL2[11-13, 19]</td></tr>
<tr><td></td><td></td><td></td><td></td><td>AtCHYR3</td><td>AT1G74770</td><td>III</td><td>BTSL1[11-13, 19]</td></tr>
<tr><td></td><td></td><td></td><td></td><td>AtCHYR4</td><td>AT3G18290</td><td>III</td><td>BTS[11-13, 19]</td></tr>
<tr><td></td><td></td><td></td><td></td><td>AtCHYR5</td><td>AT3G62970</td><td>II</td><td></td></tr>
<tr><td></td><td></td><td></td><td></td><td>AtCHYR6</td><td>AT5G18650</td><td>II</td><td>MIEL1[8, 15, 16]</td></tr>
<tr><td></td><td></td><td></td><td></td><td>AtCHYR7</td><td>AT5G25560</td><td>I</td><td></td></tr>
<tr><td></td><td>Glycine max</td><td>Gma</td><td>G. max</td><td>GmCHYR1</td><td>Glyma.03G185700</td><td>I</td><td></td></tr>
<tr><td></td><td></td><td></td><td></td><td>GmCHYR2</td><td>Glyma.03G209900</td><td>II</td><td></td></tr>
<tr><td></td><td></td><td></td><td></td><td>GmCHYR3</td><td>Glyma.05G237500</td><td>III</td><td></td></tr>
<tr><td></td><td></td><td></td><td></td><td>GmCHYR4</td><td>Glyma.06G074300</td><td>I</td><td></td></tr>
<tr><td></td><td></td><td></td><td></td><td>GmCHYR5</td><td>Glyma.07G093700</td><td>III</td><td></td></tr>
<tr><td></td><td></td><td></td><td></td><td>GmCHYR6</td><td>Glyma.07G250900</td><td>II</td><td></td></tr>
<tr><td></td><td></td><td></td><td></td><td>GmCHYR7</td><td>Glyma.08G044700</td><td>III</td><td></td></tr>
<tr><td></td><td></td><td></td><td></td><td>GmCHYR8</td><td>Glyma.09G115100</td><td>III</td><td></td></tr>
<tr><td></td><td></td><td></td><td></td><td>GmCHYR9</td><td>Glyma.09G182600</td><td>III</td><td></td></tr>
<tr><td></td><td></td><td></td><td></td><td>GmCHYR10</td><td>Glyma.11G192900</td><td>I</td><td></td></tr>
<tr><td></td><td></td><td></td><td></td><td>GmCHYR11</td><td>Glyma.13G215600</td><td>II</td><td></td></tr>
<tr><td></td><td></td><td></td><td></td><td>GmCHYR12</td><td>Glyma.14G130700</td><td>I</td><td></td></tr>
<tr><td></td><td></td><td></td><td></td><td>GmCHYR13</td><td>Glyma.15G097300</td><td>II</td><td></td></tr>
<tr><td></td><td></td><td></td><td></td><td>GmCHYR14</td><td>Glyma.17G023400</td><td>II</td><td></td></tr>
<tr><td></td><td></td><td></td><td></td><td>GmCHYR15</td><td>Glyma.17G096900</td><td>III</td><td></td></tr>
<tr><td></td><td></td><td></td><td></td><td>GmCHYR16</td><td>Glyma.17G202700</td><td>I</td><td></td></tr>
<tr><td>Monocots</td><td>Zea mays</td><td>Zma</td><td>Z. mays</td><td>ZmCHYR1</td><td>Zm00008a000345</td><td>I</td><td></td></tr>
<tr><td></td><td></td><td></td><td></td><td>ZmCHYR2</td><td>Zm00008a001478</td><td>II</td><td></td></tr>
<tr><td></td><td></td><td></td><td></td><td>ZmCHYR3</td><td>Zm00008a004267</td><td>I</td><td></td></tr>
<tr><td></td><td></td><td></td><td></td><td>ZmCHYR4</td><td>Zm00008a013953</td><td>I</td><td></td></tr>
<tr><td></td><td></td><td></td><td></td><td>ZmCHYR5</td><td>Zm00008a026365</td><td>III</td><td></td></tr>
<tr><td></td><td></td><td></td><td></td><td>ZmCHYR6</td><td>Zm00008a036783</td><td>II</td><td></td></tr>
<tr><td></td><td>Oryza sativa</td><td>Osa</td><td>O. sativa</td><td>OsCHYR1</td><td>LOC_Os01g49470</td><td>III</td><td>OsHRZ1[33]</td></tr>
<tr><td></td><td></td><td></td><td></td><td>OsCHYR2</td><td>LOC_Os01g52110</td><td>I</td><td>OsRZFP34[10]</td></tr>
<tr><td></td><td></td><td></td><td></td><td>OsCHYR3</td><td>LOC_Os03g05270</td><td>I</td><td></td></tr>
<tr><td></td><td></td><td></td><td></td><td>OsCHYR4</td><td>LOC_Os03g22680</td><td>II</td><td></td></tr>
<tr><td></td><td></td><td></td><td></td><td>OsCHYR5</td><td>LOC_Os05g47780</td><td>III</td><td>OsHRZ2[33]</td></tr>
<tr><td></td><td></td><td></td><td></td><td>OsCHYR6</td><td>LOC_Os10g31850</td><td>I</td><td></td></tr>
<tr><td></td><td></td><td></td><td></td><td>OsCHYR7</td><td>LOC_Os12g35320</td><td>II</td><td></td></tr>
<tr><td></td><td>Ananas comosus</td><td>Aco</td><td>A. comosus</td><td>AcoCHYR1</td><td>Aco002414</td><td>II</td><td></td></tr>
<tr><td></td><td></td><td></td><td></td><td>AcoCHYR2</td><td>Aco014646</td><td>I</td><td></td></tr>
<tr><td></td><td></td><td></td><td></td><td>AcoCHYR3</td><td>Aco026591</td><td>III</td><td></td></tr>
<tr><td></td><td></td><td></td><td></td><td>AcoCHYR4</td><td>Aco027553</td><td>II</td><td></td></tr>
<tr><td></td><td>Musa acuminata</td><td>Musac</td><td>M. acuminata</td><td>MusaCHYR1</td><td>GSMUA_Achr1G21090_001</td><td>III</td><td></td></tr>
<tr><td></td><td></td><td></td><td></td><td>MusaCHYR2</td><td>GSMUA_Achr8G12910_001</td><td>I</td><td></td></tr>
<tr><td></td><td></td><td></td><td></td><td>MusaCHYR3</td><td>GSMUA_Achr8G21960_001</td><td>II</td><td></td></tr>
<tr><td></td><td></td><td></td><td></td><td>MusaCHYR4</td><td>GSMUA_Achr9G19220_001</td><td>III</td><td></td></tr>
<tr><td></td><td></td><td></td><td></td><td>MusaCHYR5</td><td>GSMUA_AchrUn_randomG18350_00</td><td>III</td><td></td></tr>
<tr><td></td><td>Spirodela polyrhiza</td><td>Spipo</td><td>S. polyrhiza</td><td>SpipCHYR1</td><td>Spipo1G0018600</td><td>I</td><td></td></tr>
<tr><td></td><td>Zostera marina</td><td>Zosma</td><td>Z. marina</td><td>ZosmaCHYR1</td><td>Zosma15g00330</td><td>III</td><td></td></tr>
<tr><td></td><td></td><td></td><td></td><td>ZosmaCHYR2</td><td>Zosma211g00160</td><td>II</td><td></td></tr>
<tr><td></td><td></td><td></td><td></td><td>ZosmaCHYR3</td><td>Zosma269g00170</td><td>III</td><td></td></tr>
<tr><td>Basal angiosperms</td><td>Amborella trichopoda</td><td>Ambtr</td><td>A. trichopoda</td><td>AmbtrCHYR1</td><td>evm_27.TU.AmTr_v1.0_scaffold00057</td><td>II</td><td></td></tr>
<tr><td></td><td></td><td></td><td></td><td>AmbtrCHYR2</td><td>evm_27.TU.AmTr_v1.0_scaffold00142</td><td>I</td><td></td></tr>
<tr><td></td><td></td><td></td><td></td><td>AmbtrCHYR3</td><td>evm_27.TU.AmTr_v1.0_scaffold00079</td><td>III</td><td></td></tr>
<tr><td>Gymnosperm</td><td>Pinus parviflora</td><td>Ppar</td><td>P. parviflora</td><td>PparCHYR1</td><td>gnl|onekp|IIOL_scaffold_2011792</td><td>III</td><td></td></tr>
<tr><td></td><td></td><td></td><td></td><td>PparCHYR2</td><td>gnl|onekp|IIOL_scaffold_2077740</td><td>I</td><td></td></tr>
<tr><td></td><td></td><td></td><td></td><td>PparCHYR3</td><td>gnl|onekp|IIOL_scaffold_2003860</td><td>I</td><td></td></tr>
<tr><td></td><td></td><td></td><td></td><td>PparCHYR4</td><td>gnl|onekp|IIOL_scaffold_2003859</td><td>I</td><td></td></tr>
<tr><td></td><td></td><td></td><td></td><td>PparCHYR5</td><td>gnl|onekp|IIOL_scaffold_2010644</td><td>I</td><td></td></tr>
<tr><td></td><td>Pinus radiata</td><td>Pra</td><td>P. radiata</td><td>PraCHYR1</td><td>gnl|onekp|DZQM_scaffold_2056468</td><td>III</td><td></td></tr>
<tr><td></td><td></td><td></td><td></td><td>PraCHYR2</td><td>gnl|onekp|DZQM_scaffold_2002476</td><td>I</td><td></td></tr>
<tr><td></td><td></td><td></td><td></td><td>PraCHYR3</td><td>gnl|onekp|DZQM_scaffold_2003446</td><td>I</td><td></td></tr>
<tr><td></td><td></td><td></td><td></td><td>PraCHYR4</td><td>gnl|onekp|DZQM_scaffold_2003445</td><td>I</td><td></td></tr>
<tr><td></td><td></td><td></td><td></td><td>PraCHYR5</td><td>gnl|onekp|DZQM_scaffold_2055083</td><td>I</td><td></td></tr>
<tr><td></td><td>Pinus jeffreyi</td><td>Pje</td><td>P. jeffreyi</td><td>PjeCHYR1</td><td>gnl|onekp|MFTM_scaffold_2083811</td><td>III</td><td></td></tr>
<tr><td></td><td></td><td></td><td></td><td>PjeCHYR2</td><td>gnl|onekp|MFTM_scaffold_2012090</td><td>I</td><td></td></tr>
<tr><td></td><td></td><td></td><td></td><td>PjeCHYR3</td><td>gnl|onekp|MFTM_scaffold_2005364</td><td>I</td><td></td></tr>
<tr><td></td><td></td><td></td><td></td><td>PjeCHYR4</td><td>gnl|onekp|MFTM_scaffold_2005363</td><td>I</td><td></td></tr>
<tr><td></td><td></td><td></td><td></td><td>PjeCHYR5</td><td>gnl|onekp|MFTM_scaffold_2001893</td><td>I</td><td></td></tr>
<tr><td></td><td>Pinus ponderosa</td><td>Ppon</td><td>P. ponderosa</td><td>PponCHYR1</td><td>gnl|onekp|JBND_scaffold_2012013</td><td>III</td><td></td></tr>
<tr><td></td><td></td><td></td><td></td><td>PponCHYR2</td><td>gnl|onekp|JBND_scaffold_2011592</td><td>I</td><td></td></tr>
<tr><td></td><td></td><td></td><td></td><td>PponCHYR3</td><td>gnl|onekp|JBND_scaffold_2008589</td><td>I</td><td></td></tr>
<tr><td></td><td></td><td></td><td></td><td>PponCHYR4</td><td>gnl|onekp|JBND_scaffold_2003399</td><td>I</td><td></td></tr>
<tr><td></td><td></td><td></td><td></td><td>PponCHYR5</td><td>gnl|onekp|JBND_scaffold_2000453</td><td>I</td><td></td></tr>
<tr><td></td><td>Picea engelmanii</td><td>Pen</td><td>P. engelmanii</td><td>PenCHYR1</td><td>gnl|onekp|AWQB_scaffold_2002663</td><td>I</td><td></td></tr>
<tr><td></td><td></td><td></td><td></td><td>PenCHYR2</td><td>gnl|onekp|AWQB_scaffold_2054279</td><td>I</td><td></td></tr>
<tr><td></td><td></td><td></td><td></td><td>PenCHYR3</td><td>gnl|onekp|AWQB_scaffold_2011458</td><td>I</td><td></td></tr>
<tr><td>Pteridophyta</td><td>Selaginella moellendorffii</td><td>Sm</td><td>S. moellendorffii</td><td>SmCHYR1</td><td>403293</td><td>III</td><td></td></tr>
<tr><td></td><td></td><td></td><td></td><td>SmCHYR2</td><td>75561</td><td>III</td><td></td></tr>
<tr><td></td><td></td><td></td><td></td><td>SmCHYR3</td><td>78412</td><td>I</td><td></td></tr>
<tr><td>Bryophyta</td><td>Marchantia polymorpha</td><td>Mapoly</td><td>M. polymorpha</td><td>MapCHYR1</td><td>Mapoly0003s0292</td><td>I</td><td></td></tr>
<tr><td></td><td></td><td></td><td></td><td>MapCHYR2</td><td>Mapoly0021s0025</td><td>III</td><td></td></tr>
<tr><td></td><td>Physcomitrella patens</td><td>Pp</td><td>P. patens</td><td>PpCHYR1</td><td>Pp3c10_4820</td><td>I</td><td></td></tr>
<tr><td></td><td></td><td></td><td></td><td>PpCHYR2</td><td>Pp3c11_6970</td><td>I</td><td></td></tr>
<tr><td></td><td></td><td></td><td></td><td>PpCHYR3</td><td>Pp3c14_5600</td><td>I</td><td></td></tr>
<tr><td></td><td></td><td></td><td></td><td>PpCHYR4</td><td>Pp3c1_6070</td><td>I</td><td></td></tr>
<tr><td></td><td></td><td></td><td></td><td>PpCHYR5</td><td>Pp3c2_31820</td><td>I</td><td></td></tr>
<tr><td></td><td></td><td></td><td></td><td>PpCHYR6</td><td>Pp3c25_14500</td><td>III</td><td></td></tr>
<tr><td></td><td></td><td></td><td></td><td>PpCHYR7</td><td>Pp3c25_14501</td><td>III</td><td></td></tr>
<tr><td></td><td></td><td></td><td></td><td>PpCHYR8</td><td>Pp3c6_1960</td><td>III</td><td></td></tr>
<tr><td></td><td>Sphagnum fallax</td><td>Sfa</td><td>S. fallax</td><td>SfaCHYR1</td><td>Sphfalx0010s0273</td><td>III</td><td></td></tr>
<tr><td></td><td></td><td></td><td></td><td>SfaCHYR2</td><td>Sphfalx0011s0064</td><td>I</td><td></td></tr>
<tr><td></td><td></td><td></td><td></td><td>SfaCHYR3</td><td>Sphfalx0011s0228</td><td>I</td><td></td></tr>
<tr><td></td><td></td><td></td><td></td><td>SfaCHYR4</td><td>Sphfalx0025s0112</td><td>III</td><td></td></tr>
<tr><td></td><td></td><td></td><td></td><td>SfaCHYR5</td><td>Sphfalx0033s0070</td><td>I</td><td></td></tr>
<tr><td></td><td></td><td></td><td></td><td>SfaCHYR6</td><td>Sphfalx0055s0102</td><td>I</td><td></td></tr>
<tr><td></td><td></td><td></td><td></td><td>SfaCHYR7</td><td>Sphfalx0098s0024</td><td>I</td><td></td></tr>
<tr><td>Chlorophyta</td><td>Chlamydomonas reinhardtii</td><td>Cre</td><td>C. reinhardtii</td><td>CreCHYR1</td><td>Cre05.g248550</td><td>III</td><td></td></tr>
<tr><td></td><td></td><td></td><td></td><td>CreCHYR2</td><td>Cre17.g718300</td><td>II</td><td></td></tr>
<tr><td></td><td>Volvox carteri</td><td>Vocar</td><td>V. carteri</td><td>VocarCHYR1</td><td>Vocar.0004s0151</td><td>II</td><td></td></tr>
<tr><td></td><td></td><td></td><td></td><td>VocarCHYR2</td><td>Vocar.0019s0213</td><td>III</td><td></td></tr>
</table>
