# Supplementary material for: Genome-Wide Identification, Characterization and Expression Analysis of Soybean CHYR Gene Family
Source: Int J Mol Sci. 2021 Nov 11;22(22):12192. doi: 10.3390/ijms222212192 (PMC8625759; doi:10.3390/ijms222212192)
Supplement: Supplementary file 1 [file ijms-22-12192-s001.zip › Supplementary Table S3 Ks, Ka and KaKs calculation and divergent time of duplicated CHYR genes.pdf]

Supplementary Table S3 *Ks*, *Ka* and *Ka/Ks* calculation and divergent time of duplicated *CHYR* genes

Group	Duplicated gene pairs	*Ks*	*Ka*	*Ka/Ks*	Purify selection	Time (Mya)
I	*GmCHYR4/GmCHYR12*	0.53	0.07	0.14	Yes	43.58
I	*GmCHYR4/GmCHYR16*	0.52	0.06	0.12	Yes	42.66
I	*GmCHYR12/GmCHYR16*	0.12	0.02	0.17	Yes	9.48
II	*GmCHYR6/GmCHYR11*	0.47	0.08	0.16	Yes	38.40
II	*GmCHYR6/GmCHYR13*	0.52	0.12	0.23	Yes	42.27
II	*GmCHYR6/GmCHYR14*	0.14	0.06	0.40	Yes	11.50
II	*GmCHYR11/GmCHYR13*	0.17	0.06	0.35	Yes	13.69
II	*GmCHYR11/GmCHYR14*	0.56	0.12	0.22	Yes	46.16
II	*GmCHYR13/GmCHYR14*	0.57	0.14	0.25	Yes	46.37
III	*GmCHYR3/GmCHYR5*	0.40	0.08	0.20	Yes	33.02
III	*GmCHYR3/GmCHYR7*	0.06	0.02	0.34	Yes	5.17
III	*GmCHYR3/GmCHYR8*	1.97	0.27	0.14	Yes	161.23
III	*GmCHYR3/GmCHYR9*	0.40	0.08	0.20	Yes	32.41
III	*GmCHYR5/GmCHYR7*	0.45	0.08	0.18	Yes	37.21
III	*GmCHYR5/GmCHYR8*	1.90	0.28	0.15	Yes	155.62
III	*GmCHYR5/GmCHYR9*	0.07	0.01	0.19	Yes	5.77
III	*GmCHYR7/GmCHYR8*	1.93	0.27	0.14	Yes	158.18
III	*GmCHYR7/GmCHYR9*	0.46	0.08	0.18	Yes	37.38
III	*GmCHYR8/GmCHYR9*	1.99	0.28	0.14	Yes	162.93
III	*AtCHYR2/AtCHYR3*	0.70	0.17	0.24	Yes	23.28

Note: Mya is short for million years ago.
