# Supplementary figures and images for: Genome-Wide Identification, Characterization and Expression Analysis of Soybean CHYR Gene Family
Source: Int J Mol Sci. 2021 Nov 11;22(22):12192. doi: 10.3390/ijms222212192 (PMC8625759; doi:10.3390/ijms222212192)

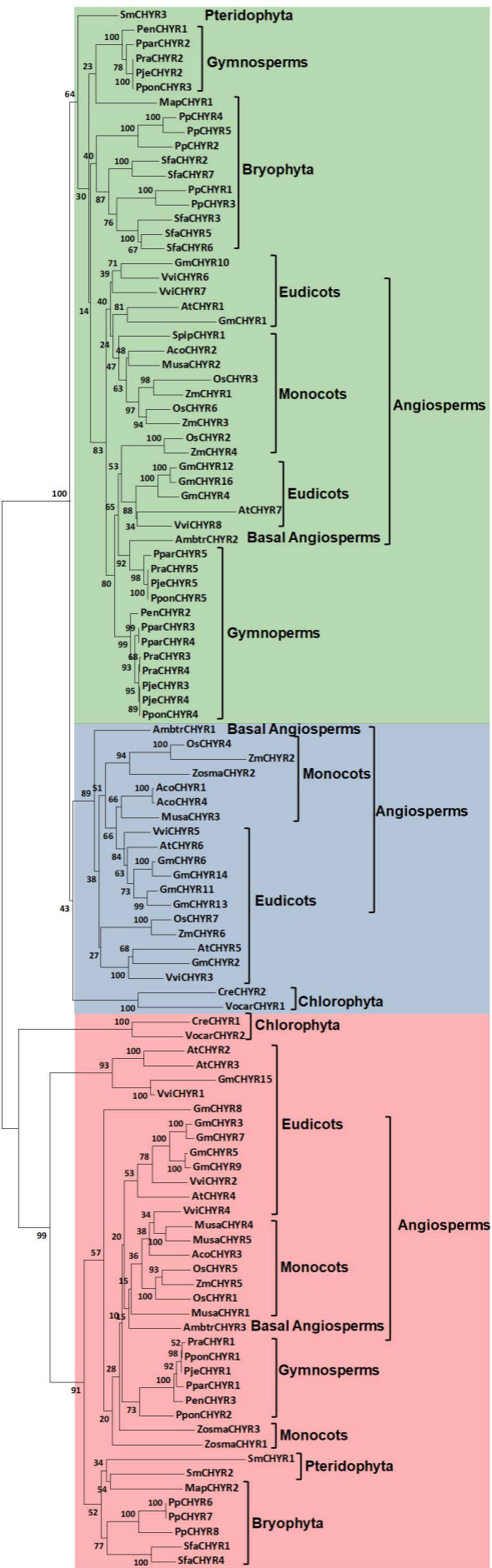

0.10

Supplement: Supplementary file 1 [file ijms-22-12192-s001.zip › Supplementary Figure S2.pdf]

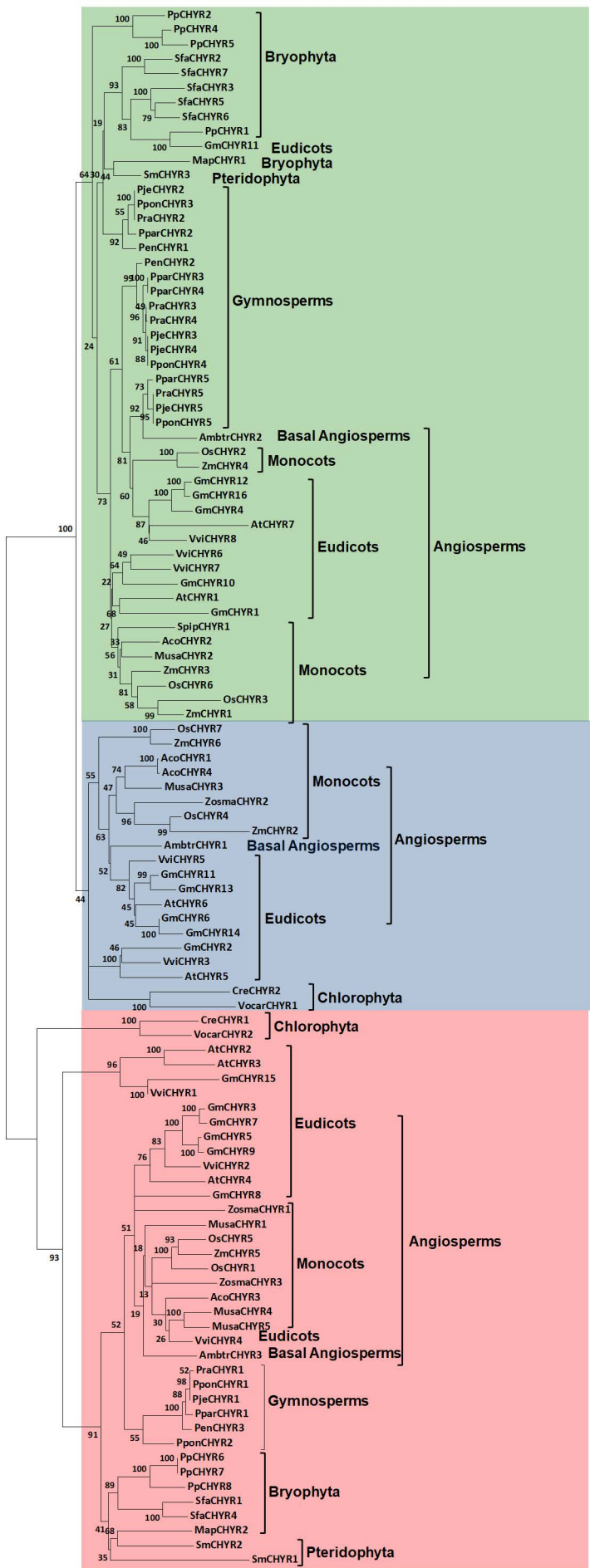

0.10

Supplement: Supplementary file 1 [file ijms-22-12192-s001.zip › Supplementary Figure S3.pdf]
